# Resistance to ERK1/2 pathway inhibitors; sweet spots, fitness deficits and drug addiction

**DOI:** 10.20517/cdr.2019.14

**Published:** 2019-06-19

**Authors:** Matthew J. Sale, Kathryn Balmanno, Simon J. Cook

**Affiliations:** Signalling Programme, The Babraham Institute, Babraham Research Campus, Cambridge CB22 3AT, UK.

**Keywords:** BRAF, *CDKN1C*/p57^KIP2^, EMT, ERK, KRAS, MEK, MEK inhibitor, resistance, selumetinib

## Abstract

MEK1/2 inhibitors are clinically approved for the treatment of BRAF-mutant melanoma, where they are used in combination with BRAF inhibitors, and are undergoing evaluation in other malignancies. Acquired resistance to MEK1/2 inhibitors, including selumetinib (AZD6244/ARRY-142866), can arise through amplification of BRAF^V600E^ or KRAS^G13D^ to reinstate ERK1/2 signalling. We have found that BRAF^V600E^ amplification and selumetinib resistance are fully reversible following drug withdrawal. This is because resistant cells with BRAF^V600E^ amplification become addicted to selumetinib to maintain a precise level of ERK1/2 signalling (2%-3% of total ERK1/2 active), that is optimal for cell proliferation and survival. Selumetinib withdrawal drives ERK1/2 activation outside of this critical “sweet spot” (~20%-30% of ERK1/2 active) resulting in a p57^KIP2^-dependent G1 cell cycle arrest and senescence or expression of NOXA and cell death with features of autophagy; these terminal responses select against cells with amplified BRAF^V600E^. ERK1/2-dependent p57^KIP2^ expression is required for loss of BRAF^V600E^ amplification and determines the rate of reversal of selumetinib resistance. Growth of selumetinib-resistant cells with BRAF^V600E^ amplification as tumour xenografts also requires the presence of selumetinib to “clamp” ERK1/2 activity within the sweet spot. Thus, BRAF^V600E^ amplification confers a selective disadvantage or “fitness deficit” during drug withdrawal, providing a rationale for intermittent dosing to forestall resistance. Remarkably, selumetinib resistance driven by KRAS^G13D^ amplification/upregulation is not reversible. In these cells ERK1/2 reactivation does not inhibit proliferation but drives a ZEB1-dependent epithelial-to-mesenchymal transition that increases cell motility and promotes resistance to traditional chemotherapy agents. Our results reveal that the emergence of drug-addicted, MEKi-resistant cells, and the opportunity this may afford for intermittent dosing schedules (“drug holidays”), may be determined by the nature of the amplified driving oncogene (BRAF^V600E^ *vs*. KRAS^G13D^), further exemplifying the difficulties of targeting KRAS mutant tumour cells.

## Introduction

The RAS-RAF-MEK1/2-ERK1/2 signalling pathway is deregulated in a variety of cancers due to mutations in pathway components, most notably BRAF and the RAS isoforms. Consequently this pathway has been the focus of major drug discovery efforts and numerous small molecule inhibitors of RAF, MEK1/2 or ERK1/2 kinase activities have been developed. Several of these have proven successful in the clinic, including the MEK1/2 inhibitors (MEKi) trametinib and cobimetinib, and the BRAF inhibitors (BRAFi) vemurafenib and dabrafenib, all of which are approved for the treatment of BRAF^V600E/K^-mutant melanoma^[1,2]^. Various other MEKi are in later stage clinical trials, including selumetinib (AZD6244/ARRY-142886) which is in phase III clinical trials^[2-5]^. MEKi are exquisitely selective because they bind within an allosteric pocket adjacent to the catalytic site that is unique to MEK1 and MEK2. MEKi also inhibit ERK1/2 signalling in RAS-mutant or wild type cells, whereas BRAFi actually promote pathway activation in these contexts and only inhibit ERK1/2 in BRAF-mutant cells^[1,6]^. Therefore MEKi have broader utility, but a narrower therapeutic margin, than BRAFi.

As with all current targeted cancer therapeutics, MEKi efficacy is limited by innate and acquired resistance and we have contributed to the understanding of both modes of MEKi resistance in colorectal cancer (CRC) cells, where *BRAF* and *KRAS* mutations are common oncogenic drivers. For example, innate resistance to MEKi is driven by strong PI3K-PKB signalling^[7]^. CRC cells with *BRAF* or *KRAS* mutations evolve resistance to MEK1/2 inhibitors by amplifying their mutant *BRAF* or *KRAS* alleles, or through emergent mutations in *MEK1*^[8-11]^. Amplification of the driving *BRAF* or *KRAS* oncogene results in overexpression of the respective oncoprotein, which in turn causes hyperphosphorylation and activation of MEK1/2. This enlarged pool of active MEK1/2, although restrained by the presence of MEKi, is sufficient to reinstate ERK1/2 phosphorylation and activation to overcome these inhibitors. Indeed, the levels of ERK1/2 phosphorylation and pathway output are reinstated to precisely that seen in parental, drug-naïve levels. Thus CRC cells evolve resistance to MEKi through profound upstream pathway activation that sufficiently overcomes the presence of MEKi to maintain ERK1/2 activity and drive proliferation and survival. A consequence of this mechanism of resistance is that in the absence of MEKi the large pool of p-MEK1/2 is no longer restrained and so MEKi withdrawal promotes rapid and sustained ERK1/2 hyperphosphorylation^[9,11]^.

Whilst moderate ERK1/2 activity is a well-established pro-proliferative and pro-survival signal^[12,13]^, excessive ERK1/2 signalling can trigger tumour suppressive mechanisms that ultimately lead to cell cycle arrest, senescence and/or cell death^[12,14]^. Cell cycle arrest in response to high RAF activity has been shown to be dependent on the cyclin-dependent kinase inhibitor (CDKI) p21^CIP1[15,16]^; indeed, ERK1/2 can promote *CDKN1A* (encodes p21^CIP1^) transcription by activating ETS and C/EBP transcription factors and promoting their binding to multiple elements within a *CDKN1A* enhancer^[17]^. Oncogenic RAS and RAF can also promote irreversible cell cycle arrest or oncogene-induced senescence (OIS) that has been shown to be dependent on ERK1/2 signalling, as well as p38 activity^[18-20]^. RAS-induced OIS is typically associated with, and often dependent upon, upregulation of p14^ARF^, p16^INK4A^, p21^CIP1^ and/or p53^[18,21-23]^.

ERK1/2 hyperactivation can also initiate or contribute to apoptotic cell death in some contexts^[14]^. Mechanisms include upregulation of death receptor ligands, such as TNF and FASL, or the death receptors themselves, including FAS, DR4 and DR5, which promote the extrinsic pathway of apoptosis^[24-28]^.

In this commentary we discuss results from our recent study^[11]^, including a novel tumour suppressive pathway activated by excessive ERK1/2 signalling involving expression of the CDKI p57^KIP2^, encoded by *CDKN1C*. p57^KIP2^ expression is strongly linked to the magnitude of ERK1/2 signalling and drives cell cycle arrest when MEKi is withdrawn from MEKi-resistant cells with BRAF^V600E^ amplification^[11]^. Excessive ERK1/2 signalling also drove the expression of the pro-apoptotic protein NOXA, and promoted apoptotic, and potentially also autophagic, cell death^[11]^. These pathways ultimately select against cells with BRAF^V600E^ amplification, thereby driving the reversibility of MEKi resistance^[11]^. In contrast MEKi-resistant cells with KRAS^G13D^ amplification do not exhibit a fitness deficit or reversal of resistance when MEKi is withdrawn, but instead undergo epithelial-to-mesenchymal transition (EMT) and exhibit chemoresistance^[11]^. These new insights may be relevant to the notion of “drug holidays” and intermittent drug dosing schedules.

## MEK1/2 inhibitor-resistant CRC cells with BRAF^V600E^ amplification are drug addicted

**Figure 1:**
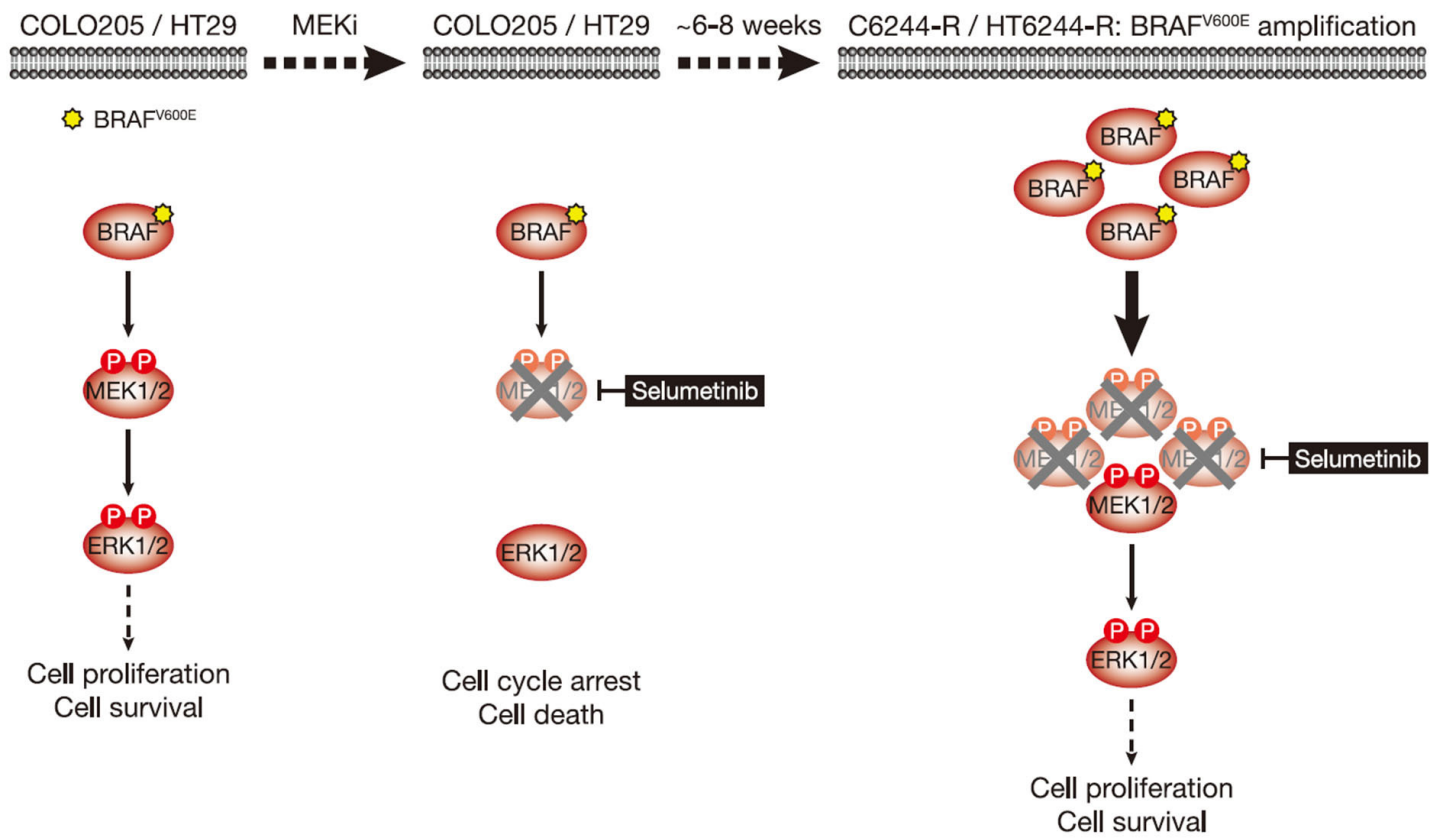
COLO205 and HT29 cells acquire resistance to the MEKi selumetinib by amplifying their driving oncogene BRAF^V600E^. COLO205 and HT29 colorectal cancer cells (both BRAF^V600E^-mutant) are addicted to ERK1/2 signalling (red) for proliferation and survival (left); inhibiting this pathway with the MEKi selumetinib halts cell proliferation and initiates cell death (middle). Selumetinib inhibits MEK1/2 by constraining the kinase domain catalytic sites in an inactive conformation, thereby inhibiting phosphorylation and activation of ERK1/2. However, selumetinib does not prevent phosphorylation of MEK1/2 by RAF (middle). Following 6-8 weeks culture in the presence of selumetinib, resistant derivatives of COLO205 (C6244-R) and HT29 (HT6244-R) cells emerge that proliferate normally and harbour amplification of BRAF^V600E^ (right). The consequent increase in BRAF^V600E^ expression results in a larger pool of p-MEK1/2 with sufficient residual activity in the presence of selumetinib to reinstate ERK1/2 phosphorylation and pathway output to those in parental COLO205 or HT29 (right). P: phosphate group

**Figure 2:**
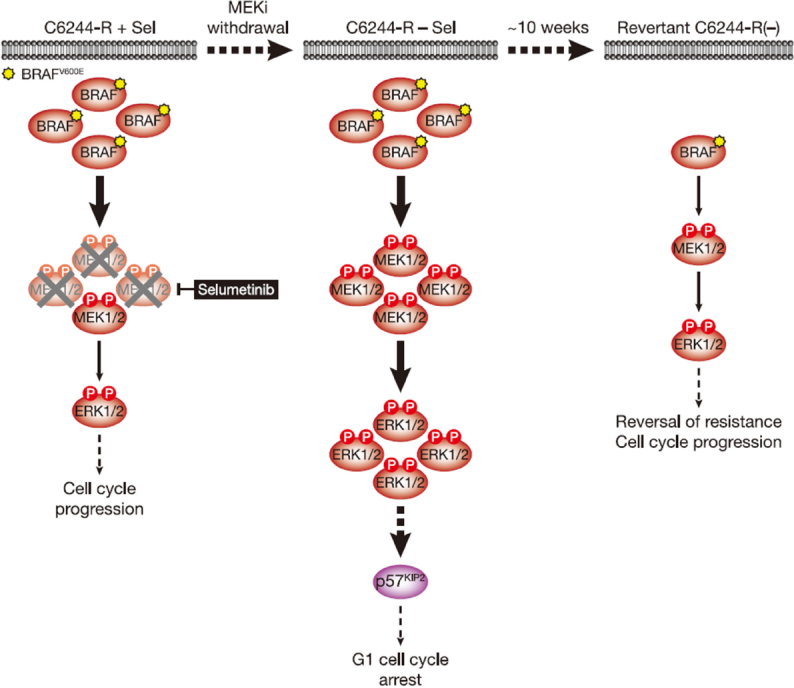
MEKi withdrawal from BRAF^V600E^-amplified C6244-R cells causes p57^KIP2^-dependent G1 cell cycle arrest and ultimately reversal of MEKi-resistance. BRAF^V600E^ amplification results in an enlarged p-MEK1/2 pool that reinstates p-ERK1/2 in selumetinib-resistant COLO205 (C6244-R) cells to parental COLO205 levels in the presence of the MEKi selumetinib (left). This level of ERK1/2 activity maintains normal cell proliferation and survival. However, when selumetinib is withdrawn (middle), this enlarged pool of p-MEK1/2 is no longer restrained and levels of p-ERK1/2 increase to ~4-5 times those in parental cells. This ERK1/2 hyperactivation drives p57^KIP2^ expression, which inhibits the cell cycle at the G1 phase (middle), and ultimately selects for reversal of selumetinib resistance (revertant C6244-R(-), right). This reversal of MEKi resistance is due to loss of BRAF^V600E^ amplification in these revertant cells and a consequent re-setting of BRAF and p-ERK1/2 back to parental COLO205 levels. P: phosphate group

BRAF^V600E^-mutant COLO205 cells acquired resistance to selumetinib by amplifying *BRAF*^*T1799A*^ (hereafter termed BRAF^V600E^ amplification)^[9]^ [Figure 1]. Parental COLO205 cells harboured three copies of chromosome 7 and *BRAF*, but following two months continuous culture in the presence of selumetinib, resistant derivatives emerged (termed C6244-R cells) that harboured 3 or 4 copies of chromosome 7 and ~10 copies of *BRAF*. Sequencing analysis revealed the selective amplification of the mutant *BRAF*^*T1799A*^ allele encoding BRAF^V600E[9]^. This amplification results in striking upregulation of BRAF protein, and 12 cell lines derived by single cell cloning of these non-clonal resistant cells exhibited remarkably similar *BRAF* levels^[11]^. In all clones, this BRAF upregulation reinstated ERK1/2 signalling in the presence of selumetinib to near-identical p-ERK1/2 levels as parental cells [Figure 1]; in contrast, when selumetinib was withdrawn all clones exhibited equivalent strong ERK1/2 hyperphosphorylation and activation of downstream targets, such as RSK, reflecting the unrestrained MEK1/2 activity arising from BRAF^V600E^ amplification [Figure 2]^[11]^.

Given that withdrawal of MEKi from resistant cells hyperactivates ERK1/2 we examined the effects of MEKi withdrawal on the stability of resistance in non-clonal and clonal selumetinib-resistant C6244-R cells seeking to define whether resistance was stable or reversible. Remarkably, reversal of selumetinib resistance was apparent within 2.5 weeks, complete in 2/3 populations by 7.5 weeks and complete in all populations by 12.5 weeks^[11]^. This reversal of MEKi resistance was accompanied by loss of BRAF upregulation and ERK1/2 phosphorylation so that both were “re-set” to parental MEKi-naïve levels [Figure 2]^[11]^. Remarkably, intrachromosomal amplification of *BRAF* was also lost; “revertant” cells derived from both non-clonal and clonal C6244-R populations harboured only 2 copies of *BRAF* and 3 copies of chromosome 7^[11]^. Given that the clonal resistant cell line harboured 4 copies of chromosome 7, two with *BRAF* amplification, this suggests that an entire chromosome 7 harbouring *BRAF* amplification was absent following reversal of resistance, whilst the *BRAF* amplicon on another was lost to yield a chromosome 7 with no copies of *BRAF*.

What are the selection pressures that drive this reversal of MEKi resistance? In the case of C6244-R cells, withdrawal of selumetinib markedly slowed proliferation and increased the fraction of cells in the G1 phase of the cell cycle^[11]^. Indeed, individual C6244-R cells with the highest p-ERK1/2 level had the lowest EdU incorporation. This G1 cell cycle arrest was maintained for at least 12 days following selumetinib withdrawal, and a subpopulation of cells exhibited features of senescence, including senescence-associated β-galactosidase activity and elevated secretion of cytokines that form part of the senescence-associated secretory phenotype^[11]^. Both proliferative arrest and senescence were ERK1/2-dependent as they could be prevented using the ERK1/2 inhibitor SCH772984^[11]^. Co-culture of COLO205 and C6244-R cells confirmed that C6244-R cells exhibited a fitness disadvantage in the absence of MEKi, with a ~35-fold enrichment of COLO205 parental cells *vs*. resistant C6244-R cells after 7 days^[11]^. This is consistent with the proliferative arrest in C6244-R being the selection pressure that drives reversal of resistance to selumetinib. As the concentration of selumetinib was increased the fitness of C6244-R increased and at concentrations > 0.1 µmol/L selumetinib C6244-R dominated the culture^[11]^.

When selumetinib was withdrawn, C6244-R cells exhibited a rapid and sustained hyperactivation of ERK1/2 far beyond the level seen in parental COLO205 cells^[9,11]^. This resulted in the anticipated induction of p21^CIP1^. However, p21^CIP1^ expression was transient, peaking 4-8 h post MEKi-withdrawal before subsiding back to basal levels^[11]^. Thus, p21^CIP1^ expression did not correlate with the sustained G1 cell cycle arrest observed following MEKi withdrawal; indeed, siRNA-mediated knock-down of p21^CIP1^ confirmed that the proliferative deficit was p21^CIP1^-independent. p21^CIP1^ expression was also low relative to other CRC cell lines, such as HCT116, possibly because p53, an important transcriptional activator of *CDKN1A*/p21^CIP1^, is mutated in COLO205 cells^[11]^. However, expression of the related CDKI p57^KIP2^ correlated well with cell cycle arrest, loss of cyclin A and p-RB following selumetinib withdrawal. Moreover, knock-out of p57^KIP2^ by CRISPR/Cas9 gene editing demonstrated that the G1 cell cycle arrest following MEKi removal was wholly dependent on p57^KIP2^. Importantly, knock-out of p57^KIP2^ also prevented or delayed reversal of resistance, demonstrating that the p57^KIP2^-dependent proliferative arrest was a key selection pressure that drove reversion to MEKi sensitivity [Figure 2]^[11]^.

**Figure 3:**
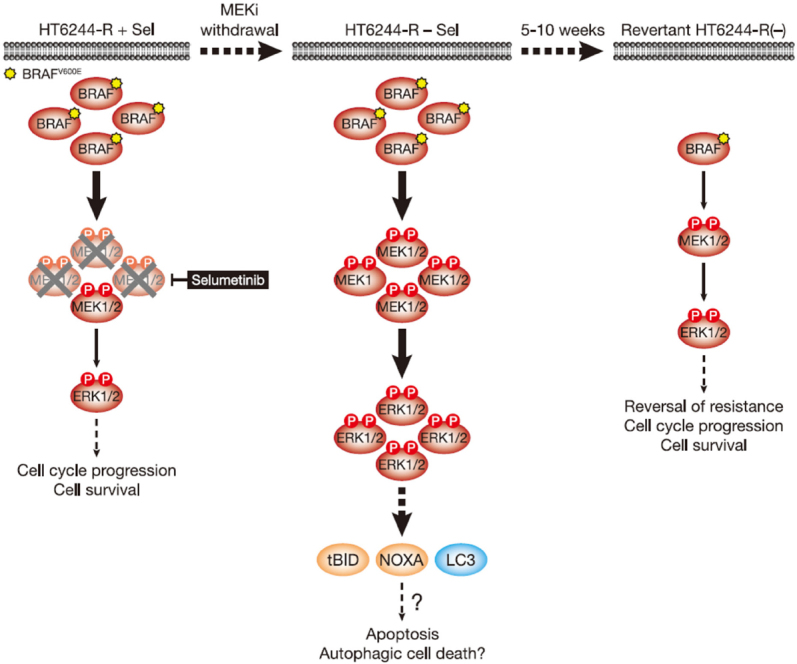
MEKi withdrawal from BRAF^V600E^-amplified HT6244-R cells causes cell death with features of apoptosis and authophagy and ultimately reversal of MEKi-resistance. BRAF^V600E^ amplification results in an enlarged p-MEK1/2 pool that reinstates p-ERK1/2 in selumetinib-resistant HT29 (HT6244-R) cells to parental HT29 levels in the presence of the MEKi selumetinib (left). This level of ERK1/2 activity maintains normal cell proliferation and survival. However, when selumetinib is withdrawn (middle), this enlarged pool of p-MEK1/2 is no longer restrained and levels of p-ERK1/2 increase to ~5 times those in parental cells. This ERK1/2 hyperactivation drives expression of pro-apoptotic NOXA and tBID, as well as processing of LC3, and cell death with features of apoptosis and autophagy. Prolonged MEKi withdrawal and cell death ultimately selects for reversal of selumetinib resistance (revertant HT6244-R(-), right). This reversal of MEKi resistance is due to loss of BRAF^V600E^ amplification in these revertant cells and a re-setting of BRAF and p-ERK1/2 back to parental HT29 levels. P: phosphate group

BRAF^V600E^-mutant HT29 cells also acquire resistance to selumetinib through *BRAF* amplification^[11]^ [Figure 1]. Again 12 clonal populations of these selumetinib-resistant HT29 (HT6244-R) cells exhibited very similar BRAF upregulation and, in the presence of selumetinib, near-identical p-ERK1/2 levels to parental HT29 cells^[11]^. Withdrawal of MEKi resulted in equivalent hyperactivation of ERK1/2 and RSK in all clones. Reversion of resistance was near-complete in some populations (clonal and non-clonal) after just 5 weeks selumetinib withdrawal and complete in all populations by 10 weeks [Figure 3]^[11]^. *BRAF* expression and p-ERK1/2 reverted to parental MEKi-naïve levels, and *BRAF* amplification was lost in these revertant cells. HT644-R clonal and non-clonal populations had 4 copies of chromosome 7, with one harbouring an intrachromosomal *BRAF* amplification, resulting in a *BRAF* copy number of 12 *vs*. 4 in parental HT29 cells^[11]^. Both clonal and non-clonal resistant cells lost *BRAF* amplification following 10 weeks MEKi withdrawal, with 5 copies of *BRAF* remaining and the chromosome 7 count maintained at 4^[11]^. Given that in these revertant cells 3 chromosomes had one copy of *BRAF* and one chromosome had 2 copies, this suggests that the amplicon was all-but lost from the chromosome with 2 copies of *BRAF* remaining. That resistance and *BRAF* amplification were reversible from clonal populations of both C6244-R and HT6244-R, and that entire chromosomes (C6244-R) or *BRAF* amplicons (C6244-R and HT644-R) were lost during reversal of resistance, supports the conclusion that loss of *BRAF* copy number was from individual cells harbouring *BRAF* amplification, rather than the result of outgrowth of rare dormant parental-like cells that persisted in the population and were selected for when MEKi was withdrawn. The cytogenetic mechanisms that underpin intrachromosomal *BRAF* amplification, and its subsequent loss, are unclear but given these data reversal of resistance must presumably involve at least slow division of cells with *BRAF* amplification.

Short-term MEKi withdrawal from HT6244-R cells caused a pronounced but transient G1 cell cycle arrest that peaked at 16 hours and returned to a near basal cell cycle profile after 72 hours^[11]^. This short-lived cell cycle disruption correlated with induction of p21^CIP1^ expression. However, from 6 days MEKi withdrawal onwards these cells underwent substantial ERK1/2-dependent cell death that was partially caspase-dependent, i.e., apoptotic [Figure 3]^[11]^. This cell death was associated with cleavage of the BH3-only protein BID to the truncated pro-apoptotic form tBID, which is a consequence of CASP8 activation following stimulation of the extrinsic pathway of apoptosis. In addition hyperactivation of ERK1/2 following MEKi removal promoted expression of the pro-apoptotic BH3-only protein NOXA. Both tBID and NOXA inhibit pro-survival BCL2 family proteins at the outer mitochondrial membrane to promote the intrinsic pathway of apoptosis. That BID cleavage occurred prior to PARP cleavage, a known CASP3 target, suggests that ERK1/2 hyperactivation triggered the extrinsic pathway of apoptosis and activation of CASP8 prior to activation of the intrinsic pathway and CASP3^[11]^. Pro-apoptotic NOXA undoubtedly contributes to activation of the intrinsic apoptotic cascade but can also promote autophagy in response to high ERK1/2 activity by binding to MCL1 and causing release of Beclin-1^[29,30]^. Indeed, MEKi withdrawal from HT6244-R cells increased processing of LC3, consistent with an upregulation of autophagy [Figure 3]^[11]^. Thus HT6244-R cells exhibited hallmarks of both apoptotic and autophagic cell death following selumetinib withdrawal, which almost certainly impose the selection pressure that ultimately drives reversal of resistance. Thus, although COLO205 and HT29 tumour cells have disabled multiple tumour suppressive mechanisms, including p53, and adapted to aberrant ERK1/2 activation arising from BRAF^V600E^ mutation, sufficient ERK1/2-responsive tumour suppressive mechanisms remain intact to drive proliferative arrest or cell death following the hyperactivation of ERK1/2 that occurs upon MEKi withdrawal.

## BRAF^V600E^-mutant CRC cells evolve to reinstate the same optimal level of ERK1/2 activity regardless of whether ERK1/2 are inhibited or hyperactivated

Cell cycle phase profile, EdU incorporation and C6244-R fitness *vs*. COLO205 were all optimal and/or maximal when C6244-R cells were maintained in 1 µmol/L selumetinib, the concentration in which they were selected and at which ERK1/2 phosphorylation matched that in parental cells^[9,11]^. However, these observations were not unique to selumetinib. C6244-R cells were cross-resistant to the clinically approved MEKis cobimetinib and trametinib, and to the ERK1/2 inhibitor SCH772984, and in each case proliferated optimally at inhibitor concentrations that imposed ERK1/2 or RSK phosphorylation at close to parental levels^[11]^. These effects were recapitulated *in vivo*: C6244-R tumours grew better in mice dosed with 10 mg/kg selumetinib compared to those dosed with vehicle only or 25 mg/kg selumetinib^[11]^. Thus, although achieving a steady-state concentration of selumetinib in mice akin to that *in vitro* is not possible, C6244-R cells were addicted to a tight window or “sweet-spot” of ERK1/2 pathway output optimal for proliferation both *in vitro* and *in vivo*.

This evolutionary pressure to restore ERK1/2 activity to an optimal “sweet-spot” was strikingly exemplified in an experiment in which separate COLO205 cell lines with resistance to a range of distinct selumetinib concentrations were established^[11]^. The higher the concentration of selumetinib, the longer the cells took to evolve resistance and proliferate normally. Remarkably, however, all resistant cells proliferated optimally in the presence of the selumetinib concentration to which they had adapted, and at this concentration exhibited equivalent p-ERK1/2 levels as parental COLO205 cells^[11]^. This was enabled by a progressive increase in *BRAF* expression: cells adapted to higher concentrations of selumetinib through higher *BRAF* expression that restored parental ERK1/2 activity and a normal cell cycle profile in the respective drug concentration^[11]^. However, in the absence of selumetinib ERK1/2 were hyperactivated in proportion to the degree of *BRAF* expression. Consequently COLO205 cells with resistance to higher concentrations of selumetinib exhibited greater ERK1/2 activation in the absence of selumetinib and underwent G1 cell cycle arrest^[11]^.

Thus regardless of whether ERK1/2 were inhibited in parental COLO205 cells, or ERK1/2 were hyperactivated following MEKi withdrawal from C6244-R cells, cells evolved accordingly to increase or decrease *BRAF* copy number and BRAF expression to a level that restored ERK1/2 activity and pathway output back to parental levels. Mass spectrometry was used to define this optimal “sweet-spot” of ERK1/2 activation; quantifying ERK1/2 activation loop dual pT-E-pY phosphorylation revealed that COLO205 cells, and C6244-R cells maintained in selumetinib, proliferated with just 2%-3% of the total ERK1/2 pool active, and cellular p-ERK1 and p-ERK2 concentrations of ~2 nmol/L and 3 nmol/L, respectively^[11]^. MEKi withdrawal increased the stoichiometry of phosphorylated ERK1/2 to ~20%-30%, and cellular p-ERK1 and p-ERK2 concentrations to ~10 and 20 nmol/L, respectively^[11]^. HT29 cells, and HT6244-R cells in selumetinib, also exhibited a ~2%-5% stoichiometry of ERK1/2 phosphorylation and cellular p-ERK1 and p-ERK2 concentrations of < 2 nmol/L and < 5 nmol/L, respectively^[11]^. This suggests, even in tumour cells with BRAF^V600E^ mutation, there is substantial spare capacity within the ERK1/2 pathway under basal conditions.

## MEK1/2 inhibitor withdrawal from KRAS-mutant CRC cells with acquired MEK1/2 inhibitor resistance promotes EMT and chemoresistance

**Figure 4:**
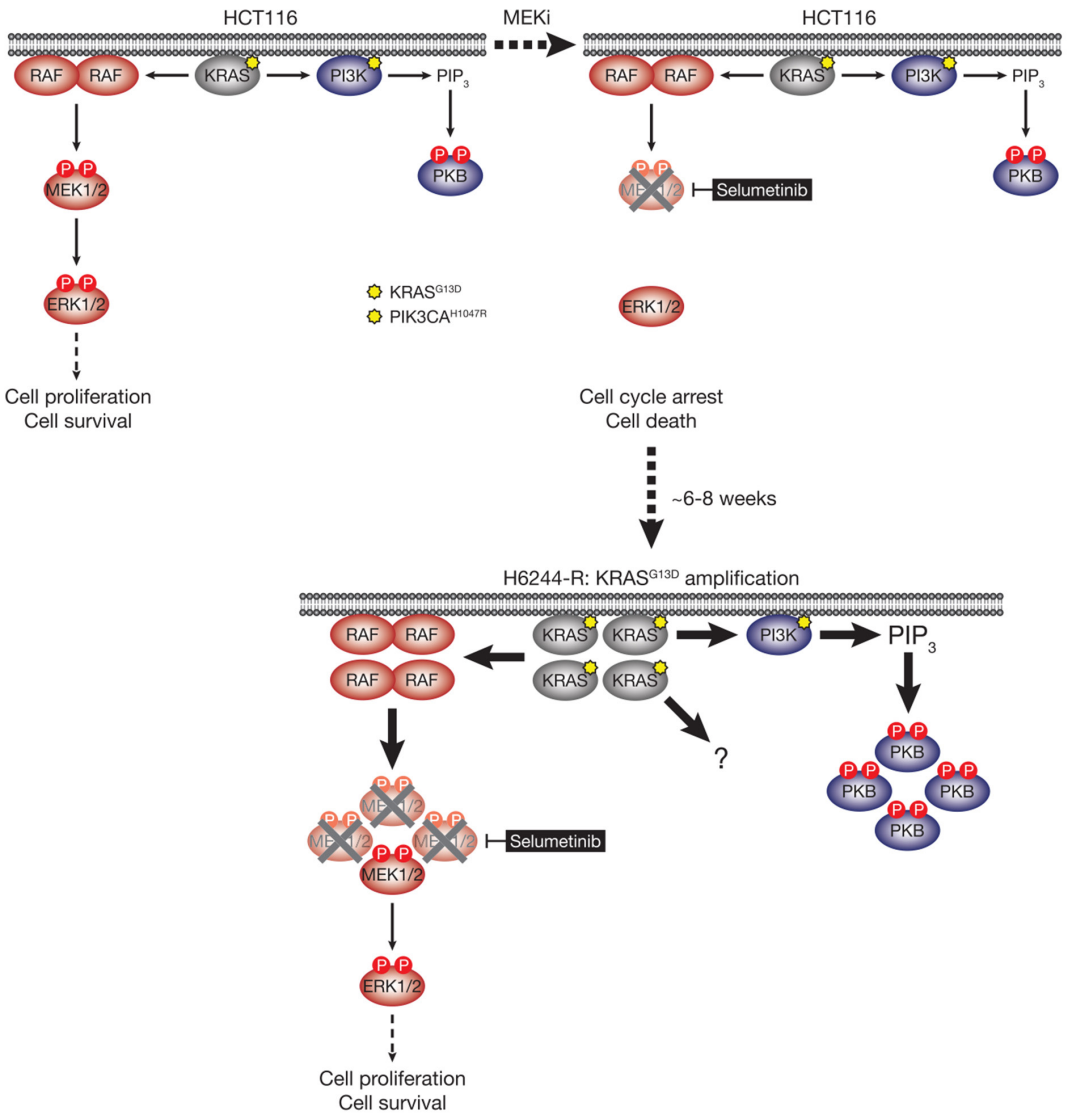
HCT116 cells acquire resistance to selumetinib by amplifying their driving oncogene KRAS^G13D^. KRAS^G13D^-mutant HCT116 cells are addicted to ERK1/2 signalling (red) for proliferation and survival (top, left); inhibiting this pathway with the MEKi selumetinib blocks cell proliferation and initiates cell death (top, right). Selumetinib inhibits MEK1/2 by constraining the kinase domain catalytic sites in an inactive conformation, thereby inhibiting phosphorylation and activation of ERK1/2 (top, right). HCT116 cells also harbour an activating H1047R mutation in *PIK3CA*, which encodes the catalytic p110α subunit of PI3K. Following 6-8 weeks culture in the presence of selumetinib, resistant derivatives of HCT116 (H6244-R) cells emerge that proliferate normally and harbour amplification of KRAS^G13D^ (bottom). The consequent increase in KRAS^G13D^ expression results in activation of a larger pool of p-MEK1/2 with sufficient residual activity in the presence of selumetinib to reinstate ERK1/2 phosphorylation and pathway activity to parental HCT116 levels (bottom). Consistent with upregulation of KRAS^G13D^, selumetinib-resistant HCT116 cells also exhibit elevated PI3K-PKB signalling (blue). P: phosphate group; PIP_3_: phosphatidylinositol-3,4,5-trisphosphate

**Figure 5:**
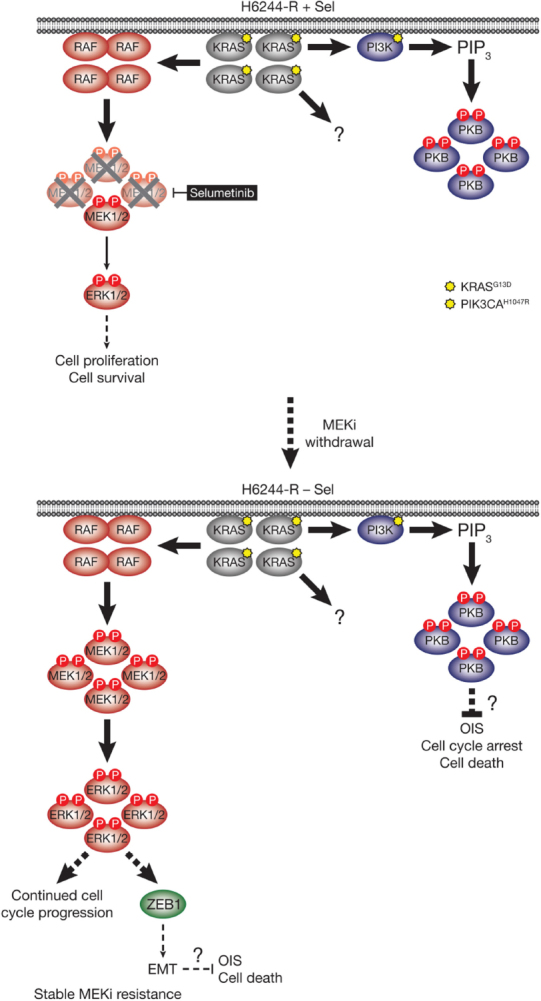
MEKi withdrawal from KRAS^G13D^-amplified H6244-R cells does not result in defective cell proliferation, cell death or reversal of resistance, but promotes a ZEB1-dependent EMT. KRAS^G13D^ amplification activates an enlarged p-MEK1/2 pool that reinstates p-ERK1/2 in selumetinib-resistant HCT116 (H6244-R) cells to parental HCT116 levels in the presence of the MEKi selumetinib (top). This level of ERK1/2 activity maintains normal cell proliferation and survival. KRAS^G13D^ amplification in these cells also drives activation of PI3K-PKB signalling. When selumetinib is withdrawn (bottom), this enlarged pool of p-MEK1/2 is no longer restrained and levels of p-ERK1/2 increase to ~5-6 times those in parental cells. ERK1/2 hyperactivation following MEKi withdrawal did not inhibit cell proliferation or induce cell death, and selumetinib-resistance was stable even after prolonged periods of drug removal. However, ERK1/2 hyperactivation drives a ZEB1-dependent epithelial-to-mesenchymal transition (EMT) that confers resistance to classic chemotherapeutics (bottom). P: phosphate group; PIP_3_: phosphatidylinositol-3,4,5-trisphosphate

**Figure 6:**
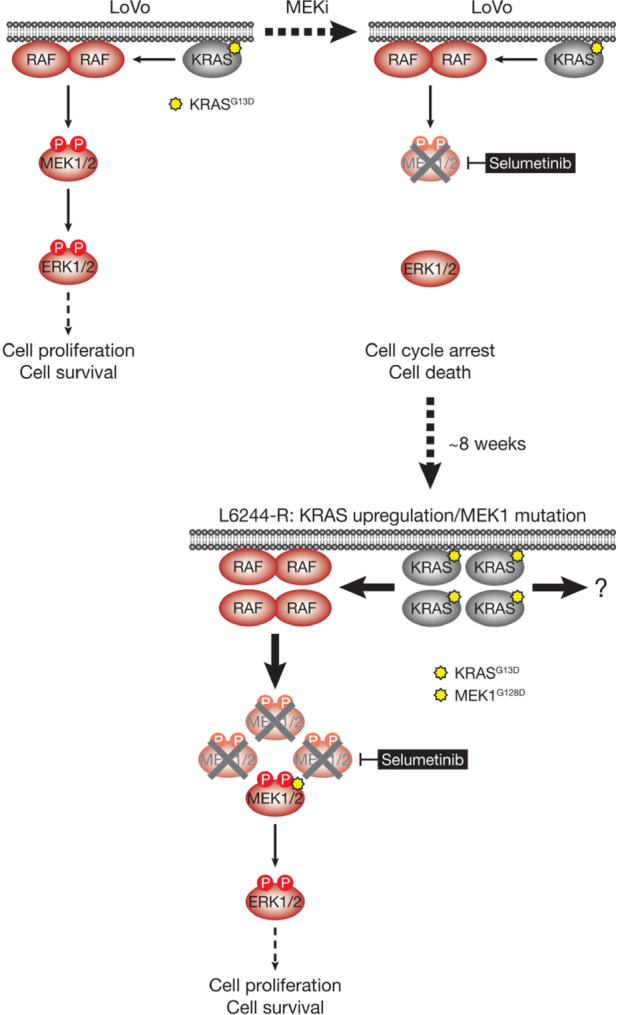
LoVo cells acquire resistance to selumetinib through KRAS^G13D^ upregulation and mutation of MEK1. KRAS^G13D^-mutant LoVo colorectal cancer cells are addicted to ERK1/2 signalling (red) for proliferation and survival (top, left); inhibiting this pathway with the MEKi selumetinib halts cell proliferation and initiates cell death. Selumetinib inhibits MEK1/2 by constraining the kinase domain catalytic sites in an inactive conformation, thereby inhibiting phosphorylation and activation of ERK1/2 (top, right). Following 6-8 weeks culture in the presence of selumetinib, resistant derivatives of LoVo (L6244-R) cells emerge that proliferate normally and exhibit upregulation of KRAS expression and MEK1^G128D^ mutation (bottom). KRAS upregulation/MEK1 mutation result in activation of a larger pool of p-MEK1/2 with sufficient residual activity in the presence of selumetinib to reinstate ERK1/2 phosphorylation and pathway output to parental LoVo levels (bottom). P: phosphate group

HCT116 CRC cells harbour a KRAS^G13D^ mutation and acquired resistance to selumetinib through *KRAS*^*G38A*^ gene amplification and striking upregulation of KRAS protein^[9]^ [Figure 4]. As with the BRAF^V600E^-amplified cells, KRAS^G13D^ amplification reinstated ERK1/2 activity and pathway output to parental levels in selumetinib resistant HCT116 (H6244-R) cells and these cells exhibited strong ERK1/2 hyperactivation following MEKi withdrawal^[9,11]^. HCT116 cells also harbour an H1047R mutation in the PI3K catalytic subunit p110α (encoded by *PIK3CA*), and unlike the BRAF^V600E^-amplified cells, KRAS^G13D^ amplification also activated PI3K-PKB signalling regardless of whether selumetinib was present [Figure 4]^[9,11]^. Remarkably, KRAS^G13D^ amplification and resistance to selumetinib were not reversible, even when drug was withdrawn for long periods (> 6 months) [Figure 5]^[11]^. In the shorter-term, these cells did not exhibit a proliferative defect, any alteration in cell cycle profile, any upregulation of CDKIs or cell death when deprived of selumetinib, and grew normally *in vivo*. In another KRAS-mutant CRC cell line, LoVo, acquired resistance to selumetinib was associated with upregulation of both the mutant and wild type *KRAS* alleles, but no change in *KRAS* copy number [Figure 6]^[11]^. In addition, several acquired mutations may contribute to MEKi resistance in these cells, including MEK1^G128D^ mutation that most likely disrupts MEKi binding^[31]^, and GNAI1^H322N^, a Giα1 subunit of heterotrimeric GTPases that may promote the activity of ERK1/2 and other signalling cascades^[32]^. Selumetinib-resistant LoVo (L6244-R) cells also exhibited parental ERK1/2 activation in the presence of selumetinib and hyperactivation in the absence of MEKi, both in non-clonal and 12 clonal derivative cell lines^[11]^. As with H6244-R, L6244-R cells also proliferated normally in the absence of MEKi, although distinct populations did exhibit different degrees of partial reversion to selumetinib sensitivity upon longer-term drug withdrawal^[11]^.

Thus the hyperactivation of ERK1/2 following MEKi withdrawal had no apparent detrimental effect on the fitness of MEKi-resistant cells with KRAS^G13D^-amplification/upregulation, which likely underlies the long-term stability of MEKi resistance in the absence of drug in these models. However, H6244-R and L6244-R cells did exhibit striking changes in cell morphology when deprived of MEKi; cells exhibited elongated protrusions, fewer cell-cell contacts, grew over one another and were more motile; all changes consistent with an epithelial-to-mesenchymal transition (EMT)^[11]^. Loss of CDH1 (E-cadherin) and increased VIM (vimentin) mRNA and protein expression confirmed that these cells had undergone an EMT, and this was associated with increased expression of SNAI1 (Snail), SNAI2 (Slug) and/or ZEB1^[11]^, transcription factors known to promote the mesenchymal phenotype and repress CDH1 transcription^[33]^. These changes following MEKi withdrawal could be prevented using the ERK1/2 inhibitor SCH772984, but not PI3K inhibitors, demonstrating that KRAS amplification acted through ERK1/2, but not PI3K, to drive EMT^[11]^. Single or double siRNA-mediated knock-down of SNAI1, SNAI2 and/or ZEB1 in H6244-R or L6244-R cells demonstrated that repression of CDH1 by ERK1/2 activation was in large part dependent on ZEB1^[11]^ [Figure 5]. Indeed, ERK2 has been shown to promote ZEB1 mRNA and protein expression and EMT in a FRA1-dependent manner^[34]^. In addition, ERK1/2 can promote recruitment of the transcriptional co-repressor CtBP to ZEB1, thereby silencing CDH1 transcription^[35]^. Although TWIST1 mRNA and protein expression are positively regulated by ERK1/2 in melanoma^[36]^, there was little change in TWIST1 mRNA or protein expression upon ERK1/2 hyperactivation in H6244-R or L6244-R cells^[11]^.

EMT has been implicated in promoting tumour invasion and metastasis^[33,37]^. However, in xenograft experiments there was no evidence of increased invasion into adjacent fat or muscle tissue when H6244-R tumours were withdrawn from selumetinib, and we could not detect liver or lung metastases in any condition^[11]^. These results may be cell line-specific or reflect the limitations of subcutaneous rather than orthotopic xenografts; attempts at orthotopic xenotransplantation were hindered by technical difficulties^[11]^. However, whilst the importance of EMT in promoting metastasis has recently been questioned, growing evidence supports a role in conferring chemoresistance^[38,39]^. Consistent with this, H6244-R cells that had undergone EMT *in vitro* were resistant to 5-fluorouracil (5-FU) and oxaliplatin, standard of care chemotherapies used to treat colorectal cancer^[11]^. L6244-R cells that had undergone EMT were also resistant to 5-FU, albeit more modestly.

## Does BRAF^V600E^ vs. KRAS^G13D^ amplification govern the distinct phenotypes of MEK1/2 inhibitor withdrawal?

As with RAF, ectopic expression of mutant RAS can inhibit proliferation and induce senescence in human fibroblasts; indeed ectopic mutant RAS expression can promote these phenotypes even in tumour cells with established endogenous RAS mutations^[21,40]^. So why was MEKi removal and ERK1/2 hyperactivation only detrimental to the BRAF^V600E^-amplified selumetinib resistant cells and not KRAS^G13D^-amplified/upregulated cells^[11]^? Are the tumour suppressive mechanisms that mitigate the oncogenic effects of excessive ERK1/2 activity still at least partially functional in some tumour cells but not others, and does BRAF^V600E^ *vs*. KRAS^G13D^ influence this? Selumetinib withdrawal caused equivalent hyperactivation of ERK1/2 in both BRAF^V600E^- and KRAS^G13D^-amplified/upregulated cells, suggesting that differences in the magnitude of ERK1/2 activation were not responsible for the contrasting consequences of MEKi removal^[11]^. One possible explanation is that the activation of other KRAS effector pathways, or KRAS and/or ERK1/2-mediated processes such as EMT, suppresses cell cycle arrest, senescence and/or cell death. Indeed, PI3K-PKB signalling downstream of mutant RAS can attenuate RAS-induced senescence^[41]^. Such a mechanism could explain why H6244-R, which exhibit striking PI3K-PKB hyperactivation, did not undergo proliferative arrest despite very high KRAS^G13D^ expression, and strong ERK1/2 hyperactivation following MEKi withdrawal^[11]^ [Figure 5]. The PI3K-PKB axis is also a well-recognised pro-proliferative and pro-survival pathway that could mitigate cell cycle arrest or pro-death effects of excessive ERK1/2 activation in H6244-R cells^[42,43]^.

Alternatively the mutational or expression status of CDKIs and/or other tumour suppressors that comprise the OIS circuitry could be an important factor. However, although mutations in key players such as p53 and CDKN2A (encodes p14ARF/p16INK4A) are present in these cell lines, their mutational and expression status did not correlate with the phenotype of MEKi withdrawal^[11]^. p53 expression was not increased by selumetinib withdrawal (at least at 72 h) in any of the BRAF^V600E^- or KRAS^G13D^-amplified/upregulated cells, and whereas COLO205 and HT29 cells harbour homozygous p53 mutations and did undergo cell cycle arrest or death, HCT116 and LoVo express wild type p53 and proliferated normally upon withdrawal of MEKi^[11]^. Whilst the CDKIs p15^INK4B^, p16^INK4A^ (mutated in HCT116), p19^INK4D^ and p21^CIP1^ may contribute to MEKi-withdrawal induced cell cycle aberrations in the BRAF^V600E^-amplified cells *vs*. the KRAS^G13D^-amplified/upregulated cells in which no CDKI upregulation occurred, their induction by ERK1/2 activation was either modest or expression levels extremely low; rather, cell cycle arrest following MEKi removal correlated with and was wholly dependent on p57^KIP2^ induction^[11]^. It is unclear why p57^KIP2^ was only regulated in this manner in C6244-R, which underwent sustained p57^KIP2^-dependent cell cycle arrest upon ERK1/2 hyperactivation; this mechanism was apparently uncoupled in HT6244-R or KRAS^G13D^-amplified/upregulated H6244-R and L6244-R cells. *CDKN1C* (encoding p57^KIP2^) is known to be silenced by methylation in many tumour types so perhaps these HT6244-R, H6244-R and L6244-R cells exemplify this^[44]^. Nevertheless, this upregulation of p57^KIP2^ represents a novel tumour suppressive mechanism by which aberrant ERK1/2 signalling inhibits proliferation and may promote senescence. Given that MEKi withdrawal increased *CDKN1C*/p57^KIP2^ mRNA expression, ERK1/2 might activate transcription of *CDKN1C*/p57^KIP2^ in a manner analogous to regulation of the closely related CDKI *CDKN1A*/p21^CIP1^. Indeed, *CDKN1C* contains several classic ERK1/2-responsive DNA-binding elements such as EGR1 and ETS^[45,46]^. Clearly, however, *CDKN1C*/p57^KIP2^ mRNA expression was not subject to the same stringent negative feedback that rapidly returned *CDKN1A*/p21^CIP1^ mRNA and protein to basal levels despite sustained ERK1/2 activation.

Whilst cell cycle arrest or cell death upon drug withdrawal was restricted to the MEKi-resistant cells with BRAF^V600E^ amplification, EMT was apparent only in KRAS^G13D^-amplified/upregulated cells despite similar hyperactivation of ERK1/2 in all cases^[11]^. MEKi withdrawal from BRAF^V600E^-amplified cells did not cause repression of CDH1, or changes in other markers of EMT. Rather, these cells expressed significantly higher levels of CDH1 than the KRAS^G13D^-amplified/upregulated cells regardless of the presence of MEKi^[11]^. This suggests that, on an epithelial-mesenchymal continuum, these BRAF^V600E^-amplified cells are more epithelial in character, consistent with the parental cell lines having epithelial (BRAF^V600E^-mutant COLO205 and HT29) or mesenchymal (KRAS^G13D^-mutant HCT116 and LoVo) EMT expression signatures^[47]^. Whether this reflects their distinct driving oncogenes, or reflects other genetic and/or epigenetic contexts that render HCT116 and LoVo cells more mesenchymal and amenable to EMT upon ERK1/2 hyperactivation is unclear. KRAS^G13D^ amplification/upregulation, through the activation of other effector pathways, may provide the required context for these cells to undergo ERK1/2-dependent EMT when MEKi is removed. PI3K-PKB signalling, which is a known promoter of EMT and is upregulated in KRAS^G13D^-amplified H6244-R cells, is an obvious candidate but was not required for repression of CDH1 upon MEKi withdrawal^[11,48]^.

Several reports have suggested that EMT can override OIS, and that ZEB1 often plays an important role^[49-51]^. ZEB1 has been suggested to suppress p15^INK4B^, p16^INK4A^ and p21^CIP1^ transcription to maintain cell proliferation^[50,51]^, though it is unclear whether ZEB1 and EMT can repress or regulate p57^KIP2^. Thus in KRAS^G13D^-amplified/upregulated H6244-R and L6244-R cells, which undergo a ZEB1-dependent EMT^[11]^, progression to OIS following MEKi withdrawal may be inhibited by EMT. EMT can also protect against apoptosis and cell death^[38,39,52]^, which is consistent with the resistance to classic chemotherapeutics exhibited by H6244-R and L6244-R following EMT. This raises the intriguing possibility that suppression of EMT when ERK1/2 are hyperactivated following MEKi removal could render H6244-R and L6244-R vulnerable to ERK1/2-driven senescence or cell death. This in turn raises the question of whether enforced EMT can protect BRAF^V600E^-amplified C6244-R or HT6244-R from proliferative arrest, senescence or cell death upon MEKi withdrawal. Thus ERK1/2 hyperactivation, in the context of KRAS^G13D^ amplification/upregulation, could mitigate its own tumour suppressive effects by triggering an EMT.

Finally, why KRAS^G13D^-mutant HCT116 and LoVo cells consistently adapt to MEKi by reinstating ERK1/2 phosphorylation and pathway output to precisely parental levels is unclear^[11]^. Evidently BRAF^V600E^-mutant COLO205 and HT29 cells must adapt by reimposing p-ERK1/2 within a narrow sweet-spot to avoid cell cycle arrest, senescence or death: clones with lower or higher p-ERK1/2 in the presence of MEKi will be out-competed by clones with parental p-ERK1/2. However, given that H6244-R and L6244-R grew normally when ERK1/2 were hyperactivated, there is no obvious selection pressure to prevent the emergence of selumetinib-resistant HCT116 and LoVo clones with higher than parental levels of p-ERK1/2^[11]^. This suggests that clones with higher levels of KRAS^G13D^ amplification/upregulation either do not arise at all, occur at some cost that is not immediately apparent and are selected against or rheostat mechanisms in the pathway maintain ERK1/2 phosphorylation at this level regardless of higher order KRAS^G13D^ amplification or expression.

## Conclusion

Our results have defined p57^KIP2^ expression as a novel tumour suppressive mechanism that responds to inappropriately activated ERK1/2. Thus, p57^KIP2^ joins p16^INK4A^ and p21^CIP1^ as ERK1/2-responsive CDKIs that mediate cell cycle arrest and/or senescence in response to high levels of ERK1/2 signalling. Our results also define p57^KIP2^ as a genetic link between high level ERK1/2 signalling and the reversibility of MEKi-resistance, suggesting that a cell autonomous ERK1/2-p57^KIP2^ pathway selects against those cells with BRAF^V600E^ amplification. Various cellular contexts probably contribute to the different phenotypes observed upon MEKi-withdrawal; for example, in BRAF^V600E^-amplified HT6244-R cells the failure to upregulate p57^KIP2^ and sustain a G1 arrest allows cells to progress instead to cell death, which also selects against BRAF^V600E^ amplification to reverse resistance. These results provide a molecular explanation, and a further rationale, for drug holidays and intermittent dosing strategies as a means of mitigating or delaying acquired resistance to ERK1/2 pathway inhibitors in cases of resistance driven by BRAF^V600E^ amplification. However, caution must be exercised in applying such strategies in the case KRAS^G13D^ amplification, where MEKi withdrawal promoted EMT, cell motility and chemoresistance, phenotypes that are highly undesirable for a drug holiday regimen. Thus reversibility of MEKi-resistance and the consequences of MEKi withdrawal may be influenced by the nature of the particular amplified oncogene - *BRAF* or *KRAS* - highlighting again the challenges of targeting cancers with KRAS mutations.

## Declarations

### Copyright

© The Author(s) 2019.

## References

[1] Holderfield M, Deuker MM, McCormick F, McMahon M (2014). Targeting RAF kinases for cancer therapy: BRAF-mutated melanoma and beyond.. Nat Rev Cancer.

[2] Caunt CJ, Sale MJ, Smith PD, Cook SJ (2015). MEK1 and MEK2 inhibitors and cancer therapy: the long and winding road.. Nat Rev Cancer.

[3] Dombi E, Baldwin A, Marcus LJ, Fisher MJ, Weiss B (2016). Activity of selumetinib in neurofibromatosis type 1-related plexiform neurofibromas.. N Engl J Med.

[4] Jänne PA, van den Heuvel MM, Barlesi F, Cobo M, Mazieres J (2017). Selumetinib plus docetaxel compared with docetaxel alone and progression-free survival in patients with KRAS-mutant advanced non-small cell lung cancer: the SELECT-1 Randomized Clinical Trial.. JAMA.

[5] Carvajal RD, Piperno-Neumann S, Kapiteijn E, Chapman PB1, Frank S (2018). Selumetinib in combination with dacarbazine in patients with metastatic uveal melanoma: a phase III, Multicenter, Randomized Trial (SUMIT).. J Clin Oncol.

[6] Poulikakos PI, Zhang C, Bollag G, Shokat KM, Rosen N (2010). RAF inhibitors transactivate RAF dimers and ERK signalling in cells with wild-type BRAF.. Nature.

[7] Balmanno K, Chell SD, Gillings AS, Hayat S, Cook SJ (2009). Intrinsic resistance to the MEK1/2 inhibitor AZD6244 (ARRY-142886) is associated with weak ERK1/2 signalling and/or strong PI3K signalling in colorectal cancer cell lines.. Int J Cancer.

[8] Corcoran RB, Dias-Santagata D, Bergethon K, Iafrate AJ, Settleman J (2010). BRAF gene amplification can promote acquired resistance to MEK inhibitors in cancer cells harboring the BRAF ^V600E^ mutation.. Sci Signal.

[9] Little AS, Balmanno K, Sale MJ, Newman S, Dry JR (2011). Amplification of the driving oncogene, KRAS or BRAF, underpins acquired resistance to MEK1/2 inhibitors in colorectal cancer cells.. Sci Signal.

[10] Sale MJ, Cook SJ (2014). Intrinsic and acquired resistance to MEK1/2 inhibitors in cancer.. Biochem Soc Trans.

[11] Sale MJ, Balmanno K, Saxena J, Ozono E, Wojdyla K (2019). MEK1/2 inhibitor withdrawal reverses acquired resistance driven by BRAF amplification but KRAS amplification drives EMT/chemoresistance.. Nat Commun.

[12] Chambard JC, Lefloch R, Pouysségur J, Lenormand P (2007). ERK implication in cell cycle regulation.. Biochim Biophys Acta.

[13] Cook SJ, Stuart K, Gilley R, Sale MJ (2017). Control of cell death and mitochondrial fission by ERK1/2 MAP kinase signalling.. FEBS J.

[14] Cagnol S, Chambard JC (2010). ERK and cell death: mechanisms of ERK-induced cell death--apoptosis, autophagy and senescence.. FEBS J.

[15] Sewing A, Wiseman B, Lloyd AC, Land H (1997). High-intensity Raf signal causes cell cycle arrest mediated by p21^Cip1^.. Mol Cell Biol.

[16] Woods D, Parry D, Cherwinski H, Bosch E, Lees E (1997). Raf-induced proliferation or cell cycle arrest is determined by the level of Raf activity with arrest mediated by p21^Cip1^.. Mol Cell Biol.

[17] Park JS, Qiao L, Gilfor D, Yang MY, Hylemon PB (2000). A role for both Ets and C/EBP transcription factors and mRNA stabilization in the MAPK-dependent increase in p21 (Cip-1/WAF1/mda6) protein levels in primary hepatocytes.. Mol Biol Cell.

[18] Lin AW, Barradas M, Stone JC, van Aelst L, Serrano M (1998). Premature senescence involving p53 and p16 is activated in response to constitutive MEK/MAPK mitogenic signaling.. Genes Dev.

[19] Zhu J, Woods D, McMahon M, Bishop JM (1998). Senescence of human fibroblasts induced by oncogenic Raf.. Genes Dev.

[20] Wang W, Chen JX, Liao R, Deng Q, Zhou JJ (2002). Sequential activation of the MEK-extracellular signal-regulated kinase and MKK3/6-p38 mitogen-activated protein kinase pathways mediates oncogenic ras-induced premature senescence.. Mol Cell Biol.

[21] Serrano M, Lin AW, McCurrach ME, Beach D, Lowe SW (1997). Oncogenic ras provokes premature cell senescence associated with accumulation of p53 and p16INK4a.. Cell.

[22] Palmero I, Pantoja C, Serrano M (1998). p19ARF links the tumour suppressor p53 to Ras.. Nature.

[23] Ferbeyre G, de Stanchina E, Lin AW, Querido E, McCurrach ME (2002). Oncogenic ras and p53 cooperate to induce cellular senescence.. Mol Cell Biol.

[24] Ulisse S, Cinque B, Silvano G, Rucci N, Biordi L (2000). Erk-dependent cytosolic phospholipase A2 activity is induced by CD95 ligand cross-linking in the mouse derived Sertoli cell line TM4 and is required to trigger apoptosis in CD95 bearing cells.. Cell Death Differ.

[25] Drosopoulos KG, Roberts ML, Cermak L, Sasazuki T, Shirasawa S (2005). Transformation by oncogenic RAS sensitizes human colon cells to TRAIL-induced apoptosis by up-regulating death receptor 4 and death receptor 5 through a MEK-dependent pathway.. J Biol Chem.

[26] Jo SK, Cho WY, Sung SA, Kim HK, Won NH (2005). MEK inhibitor, U0126, attenuates cisplatin-induced renal injury by decreasing inflammation and apoptosis.. Kidney Int.

[27] Tewari R, Sharma V, Koul N, Sen E (2008). Involvement of miltefosine-mediated ERK activation in glioma cell apoptosis through Fas regulation.. J Neurochem.

[28] Shenoy K, Wu Y, Pervaiz S (2009). LY303511 enhances TRAIL sensitivity of SHEP-1 neuroblastoma cells via hydrogen peroxide-mediated mitogen-activated protein kinase activation and up-regulation of death receptors.. Cancer Res.

[29] Sheridan C, Brumatti G, Elgendy M, Brunet M, Martin SJ (2010). An ERK-dependent pathway to Noxa expression regulates apoptosis by platinum-based chemotherapeutic drugs.. Oncogene.

[30] Elgendy M, Sheridan C, Brumatti G, Martin SJ (2011). Oncogenic Ras-induced expression of Noxa and Beclin-1 promotes autophagic cell death and limits clonogenic survival.. Mol Cell.

[31] Emery CM, Vijayendran KG, Zipser MC, Sawyer AM, Niu L (2009). MEK1 mutations confer resistance to MEK and B-RAF inhibition.. Proc Natl Acad Sci USA.

[32] Yang J, Manson DK, Marr BP, Carvajal RD (2018). Treatment of uveal melanoma: where are we now?. Ther Adv Med Oncol.

[33] Thiery JP, Acloque H, Huang RY, Nieto MA (2009). Epithelial-mesenchymal transitions in development and disease.. Cell.

[34] Shin S, Dimitri CA, Yoon SO, Dowdle W, Blenis J (2010). ERK2 but not ERK1 induces epithelial-to-mesenchymal transformation via DEF motif-dependent signaling events.. Mol Cell.

[35] Ichikawa K, Kubota Y, Nakamura T, Weng JS, Tomida T (2015). MCRIP1, an ERK substrate, mediates ERK-induced gene silencing during epithelial-mesenchymal transition by regulating the co-repressor CtBP.. Mol Cell.

[36] Weiss MB, Abel EV, Mayberry MM, Basile KJ, Berger AC (2012). TWIST1 is an ERK1/2 effector that promotes invasion and regulates MMP-1 expression in human melanoma cells.. Cancer Res.

[37] Ye X, Weinberg RA (2015). Epithelial-mesenchymal plasticity: a central regulator of cancer progression.. Trends Cell Biol.

[38] Fischer KR, Durrans A, Lee S, Sheng J, Li F (2015). Epithelial-to-mesenchymal transition is not required for lung metastasis but contributes to chemoresistance.. Nature.

[39] Zheng X, Carstens JL, Kim J, Scheible M, Kaye J (2015). Epithelial-to-mesenchymal transition is dispensable for metastasis but induces chemoresistance in pancreatic cancer.. Nature.

[40] Unni AM, Harbourne B, Oh MH, Wild S, Ferrarone JR (2018). Hyperactivation of ERK by multiple mechanisms is toxic to RTK-RAS mutation-driven lung adenocarcinoma cells.. Elife.

[41] Kennedy AL, Morton JP, Manoharan I, Nelson DM, Jamieson NB (2011). Activation of the PIK3CA/AKT pathway suppresses senescence induced by an activated RAS oncogene to promote tumorigenesis.. Mol Cell.

[42] Fruman DA, Chiu H, Hopkins BD, Bagrodia S, Cantley LC (2017). The PI3K pathway in human disease.. Cell.

[43] Manning BD, Toker A (2017). AKT/PKB signaling: navigating the network.. Cell.

[44] Kavanagh E, Joseph B (2011). The hallmarks of CDKN1C (p57, KIP2) in cancer.. Biochim Biophys Acta.

[45] Figliola R, Busanello A, Vaccarello G, Maione R (2008). Regulation of p57(KIP2) during muscle differentiation: role of Egr1, Sp1 and DNA hypomethylation.. J Mol Biol.

[46] Pateras IS, Apostolopoulou K, Niforou K, Kotsinas A, Gorgoulis VG (2009). p57KIP2: "Kip"ing the cell under control.. Mol Cancer Res.

[47] Schlicker A, Beran G, Chresta CM, McWalter G, Pritchard A (2012). Subtypes of primary colorectal tumors correlate with response to targeted treatment in colorectal cell lines.. BMC Med Genomics.

[48] Xu W, Yang Z, Lu N (2015). A new role for the PI3K/Akt signaling pathway in the epithelial-mesenchymal transition.. Cell Adh Migr.

[49] Ansieau S, Bastid J, Doreau A, Morel AP, Bouchet BP (2008). Induction of EMT by twist proteins as a collateral effect of tumor-promoting inactivation of premature senescence.. Cancer Cell.

[50] Liu Y, El-Naggar S, Darling DS, Higashi Y, Dean DC (2008). Zeb1 links epithelial-mesenchymal transition and cellular senescence.. Development.

[51] Ohashi S, Natsuizaka M, Wong GS, Michaylira CZ, Grugan KD (2010). Epidermal growth factor receptor and mutant p53 expand an esophageal cellular subpopulation capable of epithelial-to-mesenchymal transition through ZEB transcription factors.. Cancer Res.

[52] Singh A, Settleman J (2010). EMT, cancer stem cells and drug resistance: an emerging axis of evil in the war on cancer.. Oncogene.

